# Burden of Severe Pneumonia, Pneumococcal Pneumonia and Pneumonia Deaths in Indian States: Modelling Based Estimates

**DOI:** 10.1371/journal.pone.0129191

**Published:** 2015-06-18

**Authors:** Habib Farooqui, Mark Jit, David L. Heymann, Sanjay Zodpey

**Affiliations:** 1 Public Health Foundation of India, New Delhi, India; 2 London School of Hygiene and Tropical Medicine, London, United Kingdom; 3 Modelling and Economics Unit, Public Health England; University of Otago, NEW ZEALAND

## Abstract

The burden of severe pneumonia in terms of morbidity and mortality is unknown in India especially at sub-national level. In this context, we aimed to estimate the number of severe pneumonia episodes, pneumococcal pneumonia episodes and pneumonia deaths in children younger than 5 years in 2010. We adapted and parameterized a mathematical model based on the epidemiological concept of potential impact fraction developed CHERG for this analysis. The key parameters that determine the distribution of severe pneumonia episode across Indian states were state-specific under-5 population, state-specific prevalence of selected definite pneumonia risk factors and meta-estimates of relative risks for each of these risk factors. We applied the incidence estimates and attributable fraction of risk factors to population estimates for 2010 of each Indian state. We then estimated the number of pneumococcal pneumonia cases by applying the vaccine probe methodology to an existing trial. We estimated mortality due to severe pneumonia and pneumococcal pneumonia by combining incidence estimates with case fatality ratios from multi-centric hospital-based studies. Our results suggest that in 2010, 3.6 million (3.3–3.9 million) episodes of severe pneumonia and 0.35 million (0.31–0.40 million) all cause pneumonia deaths occurred in children younger than 5 years in India. The states that merit special mention include Uttar Pradesh where 18.1% children reside but contribute 24% of pneumonia cases and 26% pneumonia deaths, Bihar (11.3% children, 16% cases, 22% deaths) Madhya Pradesh (6.6% children, 9% cases, 12% deaths), and Rajasthan (6.6% children, 8% cases, 11% deaths). Further, we estimated that 0.56 million (0.49–0.64 million) severe episodes of pneumococcal pneumonia and 105 thousand (92–119 thousand) pneumococcal deaths occurred in India. The top contributors to India’s pneumococcal pneumonia burden were Uttar Pradesh, Bihar, Madhya Pradesh and Rajasthan in that order. Our results highlight the need to improve access to care and increase coverage and equity of pneumonia preventing vaccines in states with high pneumonia burden.

## Introduction

Pneumonia is one of the most common causes of morbidity and mortality in children younger than 5 years in India[1]. The Millennium Development Goal (MDG) 4, which focuses on reduction of under-five mortality[2], has generated significant momentum for accurate assessment of cause-specific under five morbidity and mortality. In 2004, global estimates on incidence of clinical pneumonia in children younger than 5 years were first generated and published by Rudan et al.[3]. For developing countries, these estimates suggested that median incidence was 0.28 episodes per child-year, with an interquartile range 0.21–0.71 episodes per child-year[3]. In 2008, the Child Health Epidemiology Reference Group (CHERG) established by the World Health Organization (WHO) revised the estimates of childhood pneumonia morbidity and mortality and also identified lack of exclusive breastfeeding, under-nutrition, indoor air pollution, low birth weight, crowding and lack of measles immunization as leading risk factors contributing to pneumonia incidence. In addition, they mentioned that five countries where 44% of the world’s children aged less than 5 years live (India, China, Pakistan, Bangladesh, Indonesia, Nigeria) contribute more than half of the new pneumonia cases annually. For India, they predicted around 43 million pneumonia cases (23% of the world’s total) and estimated an incidence of 0.37 episodes per child-year for clinical pneumonia[4].

One of the major contributors to the pneumonia burden is *Streptococcus pneumonia*; others include *Hemophilus influenzae*, *Respiratory syncitical virus* and Influenza. However, the estimation of pneumococcal pneumonia among clinical pneumonia episodes has remained a challenge in developing nations due to lack of laboratory diagnostic support and surveillance systems to capture the etiologic agents for pneumonia. Also, the majority of pneumococcal pneumonia infections are not bacteraemic, and hence not identifiable through cultures of sterile site body fluids[5]. To address these limitations and to estimate the true burden of vaccine preventable diseases, the vaccine probe approach has emerged as a promising alternative. In this approach, the burden of pneumonia prevented by a specific vaccine is presumed to be a minimum estimate of the burden of pneumonia due to the organism against which the vaccine is directed[6]. Using this approach, O’Brien et al. estimated burden of disease caused by *Streptococcus pneumoniae* in children younger than 5 years by applying efficacy estimates derived from pneumococcal vaccine trials to WHO country-specific estimates of all-cause pneumonia cases and deaths.[5]. In 2013, Walker et al. published the first ever global estimates of the contribution of other vaccine-preventable causes (*Streptococcus pneumoniae*, *Haemophilus influenzae* and influenza) of severe clinical pneumonia cases and deaths using the same approach[7].

However, one of the limitations of the above global pneumonia estimates[3–5, 7] was focus on WHO regions instead of the member states. This limitation has been addressed partly by Rudan et al.[8] in their revised estimates on incidence, severe morbidity, mortality, underlying risk factors and causative pathogens of clinical pneumonia for 192 countries. However, for a large country like India, estimates of pneumonia burden at sub-national (states) level are still not available. It is recognized that for India, which accounts for 23% of the global pneumonia burden and 36% of the WHO regional burden, national estimates may hide significant sub-national disparities[1, 4]. For example, a large nationally representative survey has reported that in 2005, pneumonia contributed 13.5% of deaths in children age 1–59 months. However, it was observed that the girls in central India had a five-times higher mortality rate (per 1000 live births) from pneumonia than did boys in south India[9]. Further, the lack of reliable estimates of pneumonia burden in terms of etiology has led to under-utilization of existing preventive interventions. In this context, we have generated Indian state-specific estimates for burden of severe pneumonia, pneumococcal pneumonia and pneumonia deaths to provide guidance for pneumonia control program.

## Methods

To estimate the number of new episodes of severe pneumonia for each Indian state we have adapted and parameterized a model that was originally developed and validated by the CHERG group[4] using Microsoft Excel. The model is based on the epidemiological concept of potential impact fraction[10] as follows:

$$Ν\text{e/cy}\;=\;\left(Pop<\text{5yrs}\right)\times \left(Inc\text{Ind}\right)\times \left\{1+\sum_{\left(RF\;=\;1\to n\right)}\left[\left(Prev\text{RFn}-Prev\text{RFnInd}\right)\times \left(RR\text{RFn}-1\right)\right]\right\}$$

where N_e/cy_ is the number of new episodes of severe pneumonia per year in each Indian state, Pop_< 5yrs_ is the population of children less than 5 years in each state, Inc_Ind_ is the estimated incidence of severe clinical pneumonia at the all-India level, Prev_RFn_ is the prevalence of exposure to n-th risk factor among under-fives in the Indian state, Prev_RFnInd_ is the prevalence of exposure to n-th risk factor among under-fives at all India level and RR_RFn_ is the relative risk for developing pneumonia associated with the n-th risk factor.

The methods and model used to estimate and distribute the annual number of severe pneumonia episodes and pneumococcal pneumonia episodes has been explained in detail in a subsequent section. In addition, the parameter values and associated data sources are also explained.

### Step 1

The total number of severe pneumonia episodes in children age less than 1 year was estimated by multiplying the number of all children age less than 1 year living in each Indian state in year 2010 by an incidence of 0.068 severe pneumonia episodes per child-year.

The data on population for each Indian state for year 2010 (with one-year age interval) was collected from Census of India[11] whereas the incidence of severe pneumonia in children age less than 1 year was estimated through meta-analysis of selected population based cohort studies[12–14] that have used WHO-defined chest-radiography-positive pneumonia in case definition. However, the incidence rate of severe pneumonia for other age intervals (1–2 years, 2–3 years, 3–4 years and 4–5 years) was estimated through fitting data from hospital based multi-centric surveillance study[15, 16] to an appropriate distribution (gamma). The incidence estimates were adjusted to account for access to health services. The information regarding access to health services was imputed from District Level Household and Facility Survey (DLHS-3) which is a nationwide survey covering 601 districts from 34 states and union territories of India[17]. Finally, the total number of severe pneumonia episodes in children age less than 5 years was calculated as sum total of all severe pneumonia episodes across all age intervals, which was estimated through repeating the process, mentioned above for other age intervals (1–2 years, 2–3 years, 3–4 years and 4–5 years) (S1 File).

### Step 2

The total number of severe pneumonia episodes in children age less than 5 years estimated from step 1 were then distributed into state-specific estimates on the basis of three parameters (a) state-specific under-5 population, (b) state-specific prevalence of selected definite pneumonia risk factors (prevalence of malnutrition (weight–for–age z<–2), low birth weight (≤2500 g), non–exclusive breastfeeding (in the first 4 months) and solid fuel use (‘yes’) and (c) meta-estimates of relative risks for each of these risk factors[4].

The information on prevalence of the definite risk factors was available through National Family Health Survey-3 (NFHS) [18]. The NFHS surveys are conducted every 5–6 years under the stewardship of the Ministry of Health and Family Welfare (MOHFW), Government of India. The survey is based on a sample of households that is representative at the national and state levels and provides trend data on key indicators. The key risk factors included in the model—i.e. prevalence of malnutrition, low birth weight, non–exclusive breastfeeding and solid fuel use—have consistently shown significant effects in multivariate study designs [4, 7, 8, 19, 20]. The meta-estimates of relative risks (RR) for the selected definite risk factors were taken from an earlier study[4] as follows: malnutrition-(RR-1.8), low birth weight (RR-1.4), non-exclusive breastfeeding (RR-1.9) and indoor air pollution (RR-1.8).

### Step 3

The total number of pneumococcal pneumonia episodes was estimated by applying attributable proportion of 36.51% for *Streptococcus pneumoniae* [5]to all-cause severe pneumonia episodes in children age less than 5 years across Indian states estimated from step 2.

To determine the proportions of pneumonia cases attributable to *Streptococcus pneumoniae*, we relied on pneumococcal conjugate vaccine efficacy trial conducted in Philippines, which was the only trial conducted in Asia[21]. This trial was conducted in 2000 prior to introduction of Hib in Philippines (2010), so prevalence of non-pneumococcal causes of pneumonia was likely to be similar. The efficacy of the vaccine against a relevant definition of pneumonia was adjusted to account for vaccine serotype coverage and efficacy against pneumococcal pneumonia. We used the conjugate vaccine efficacy against WHO-defined chest-radiography-positive pneumonia as a measure of the proportion of pneumonia cases attributable to *S pneumoniae*. The Philippines trial reported 19.8% efficacy against radiologic pneumonia ((95% CI: -8.8, 40.8) in children age less than 1 year. The 11 serotypes contained in the vaccine (1, 3, 4, 5, 6B, 7F, 9V, 14, 18C, 19F and 23F) were estimated to account for 65.33% of Invasive Pneumococcal Disease (IPD) in the India based on a multi-centric surveillance study[22]. With vaccine efficacy against vaccine-type pneumococcal disease assumed to be 83%[23], we estimated that 23.8% (= 19.8/83) of radiologic pneumonia cases are due to the 11-pneumococcal serotypes in the vaccine and 36.51% due to any pneumococcal serotypes. Hence, radiologic pneumonia cases attributable to *S Pneumoniae* were 36.51%.

### Step 4

To estimate and distribute all-cause pneumonia mortality and pneumococcal pneumonia mortality in children age less than 5 years, the case fatality rate of 1.95% in severe pneumonia was applied to all-cause severe pneumonia episodes estimated in step 2 and the case fatality rate of 16.7% in pneumococcal pneumonia was applied to pneumococcal pneumonia episodes estimated in step 3. These mortality estimates in children age less than 5 years were estimated from multi-centric hospital based studies[14, 24] and were scaled across Indian states in accordance with pneumonia mortality rates reported by Million Death Study for India[1]. The parameterized model is provided as supplementary material (S1 File).

## Results

We estimated that in year 2010, 3.6 million (3.3–3.9 million) episodes of severe pneumonia and 0.35 million (0.3–0.4 million) pneumonia deaths (all-cause) occurred in children younger than 5 years in India (Table 1). The estimated incidence of severe pneumonia was 30.7 (95% CI, 28.1–33.5) per 1000 children per year in those less than 5 years of age, and 87.3 (95% CI, 80.1–95.2) in children aged less than 1 year.

**Table 1:** Distribution of severe pneumonia and pneumococcal pneumonia episodes and deaths in children younger than 5 years in 2010, across Indian states.

	Indian State	Population aged 0–5 years	Severe Pneumonia Incidence*	Severe Pneumonia Episodes (Thousands)	Pneumonia Deaths (Thousands)	Pneumococcal Pneumonia Episodes (Thousands)	Pneumococcal Pneumonia Deaths (Thousands)
**North**	**Delhi**	1381200	8.0 (7.3–8.7)	11.0 (10.1–12.0)	0.6 (0.6–0.7)	1.7 (1.5–2.0)	0.2 (0.2–0.2)
	**Haryana**	2362519	28.0 (25.7–30.5)	66.2 (60.7–70.2)	4.8 (4.2–5.5)	10.4 (9.1–11.9)	1.4 (1.2–1.6)
	**Himachal Pradesh**	544984	18.6 (17.1–20.3)	10.1 (9.3–11.1)	0.7 (0.6–0.8)	1.6 (1.4–1.8)	0.2 (0.2–0.2)
	**Jammu & Kashmir**	1414884	17.2 (15.8–18.8)	24.4 (22.4–26.6)	2.0 (1.7–2.3)	3.8(3.4–4.4)	0.6 (0.5–0.7)
	**Punjab**	2133529	21.9 (20.1–23.9)	46.8 (42.9–51.0)	2.1 (1.8–2.4)	7.4 (6.5–8.4)	0.6 (0.5–0.7)
	**Rajasthan**	7302170	38.1 (35.0–41.6)	278.3 (255.3–303.4)	40.3 (35.2–46.1)	43.9 (38.5–50.1)	11.9 (10.4–13.6)
	**Uttaranchal**	924864	28.1 (25.8–30.6)	26.0 (23.8–28.3)	2.9 (2.5–3.3)	4.1 (3.6–4.7)	0.9 (0.7–1.0)
**Central**	**Chhattisgarh**	2541065	28.2 (25.8–30.7)	71.6 (65.7–78.1)	7.1 (6.2–8.1)	11.3 (9.9–12.9)	2.1 (1.8–2.4)
	**Madhya Pradesh**	7471286	44.3 (40.7–48.3)	331.2 (303.7–361.1)	44.2 (38.7–50.5)	52.2 (45.8–59.6)	13.4 (11.4–14.9)
	**Uttar Pradesh**	20376668	41.4 (38.0–45.2)	844.0 (774.1–920.2)	94.2 (82.4–107.7)	133.2 (116.7–151.9)	27.8 (24.4–31.7)
**East**	**Bihar**	12765029	45.5 (41.7–49.6)	580.4 (532.3–632.8)	78.6 (68.8–89.9)	91.6 (80.3–104.5)	23.2 (20.3–26.5)
	**Jharkhand**	3648297	50.3 (46.2–54.9)	183.6 (168.4–200.2)	21.3 (18.7–24.4)	29.0 (25.4–33.1)	6.3 (5.5–7.2)
	**Orissa**	3653029	36.4 (33.4–39.7)	133.0 (122.0–145.0)	11.6 (10.1–13.2)	21.0 (18.4–23.9)	3.4 (3.0–3.9)
	**West Bengal**	7333143	27.8 (25.5–30.3)	203.7 (186.8–222.1)	9.9 (8.6–11.3)	32.1 (28.2–36.7)	2.9 (2.6–3.3)
**North East**	**Arunachal Pradesh**	142879	14.5 (13.3–15.8)	2.1 (1.9–2.3)	0.1 (0.1–0.1)	0.3 (0.3–0.4)	0.0 (0.0–0.0)
	**Assam**	3212833	27.3 (25.0–29.7)	87.6 (80.3–95.5)	7.6 (6.6–8.6)	13.8 (12.1–15.8)	2.2 (2.0–2.5)
	**Manipur**	232029	26.7 (24.5–29.1)	6.2 (5.7–6.8)	0.3 (0.3–0.3)	1.0 (0.9–1.1)	0.1 (0.1–0.1)
	**Meghalaya**	406154	25.2 (23.1–27.5)	10.2 (9.4–11.2)	0.5 (0.4–0.6)	1.6 (1.4–1.8)	0.1 (0.1–0.2)
	**Mizoram**	121233	7.2 (6.6–7.8)	0.9 (0.8–0.9)	0.0 (0.0–0.0)	0.1 (0.1–0.2)	0.0 (0.0–0.0)
	**Nagaland**	197262	33.1 (30.3–36.1)	6.5 (6.0–7.1)	0.3 (0.3–0.4)	1.0 (0.9–1.2)	0.1 (0.1–0.1)
	**Sikkim**	42336	9.6 (8.8–10.5)	0.4 (0.4–0.4)	0.0 (0.0–0.0)	0.1 (0.1–0.1)	0.0 (0.0–0.0)
	**Tripura**	322566	28.8 (26.4–31.4)	9.3 (8.5–10.1)	0.5 (0.4–0.5)	1.5 (1.3–1.7)	0.1 (0.1–0.2)
**West**	**Goa**	101203	11.9 (10.9–13.0)	1.2 (1.1–1.3)	0.1 (0.1–0.1)	0.2 (0.2–0.2)	0.0 (0.0–0.0)
	**Gujarat**	5460332	26.4 (24.2–28.8)	144.1 (132.2–157.1)	8.7 (7.6–10.0)	22.7 (19.9–25.9)	2.6 (2.3–2.9)
	**Maharashtra**	9362026	16.2 (14.9–17.7)	152.0 (139.4–165.8)	6.7 (5.7–7.6)	24.0 (21.0–27.4)	2.0 (1.7–2.2)
**South**	**Andhra Pradesh**	6284765	23.2 (21.3–25.3)	145.9 (133.9–159.1)	5.5 (4.8–6.3)	23.0 (20.2–26.3)	1.6 (1.4–1.9)
	**Karnataka**	5046719	19.5 (17.8–21.2)	98.2 (90.1–107.1)	4.7 (4.1–5.4)	15.5 (13.6–17.7)	1.4 (1.2–1.6)
	**Kerala**	2453092	11.5 (10.5–12.5)	28.2 (25.9–30.7)	0.2 (0.1–0.2)	4.4 (3.9–5.1)	0.0 (0.0–0.1)
	**Tamil Nadu**	5278701	13.8 (12.7–15.1)	73.0 (66.8–79.5)	1.0 (0.8–1.1)	11.5 (10.1–13.1)	0.3 (0.3–0.3)
	**All India**	112516797	30.7 (28.1–33.5)	3576.0 (3279.8–3898.9)	356.3 (311.6–407.4)	564.2 (494.5–643.8)	105.1 (92.1–120.0)

Data in parenthesis are 95% confidence intervals

* Incidence in children aged 0–59 months per 1000 children younger than 5 years

Further, we conducted a disaggregated state-specific analysis for 29 states of India. The estimated annual severe pneumonia incidence varied greatly from one state to another: 7.2 episodes (95% CI, 6.6–7.8) per 1000 children in Manipur to 50.3 episodes (95% CI, 46.2–54.9) per 1000 children in Jharkhand. We observed that southern states (Kerala, Tamil Nadu) and northeastern states (Sikkim, Manipur) have significantly lower incidence and mortality from severe pneumonia as compared to rest of the India (Table 1). In age-stratified analysis, we observed that severe pneumonia related morbidity followed a skewed distribution: highest morbidity in the 0–1 year age group (51%) followed by the 1–2 year age group (22%) (S1 File).

**Fig 1:**
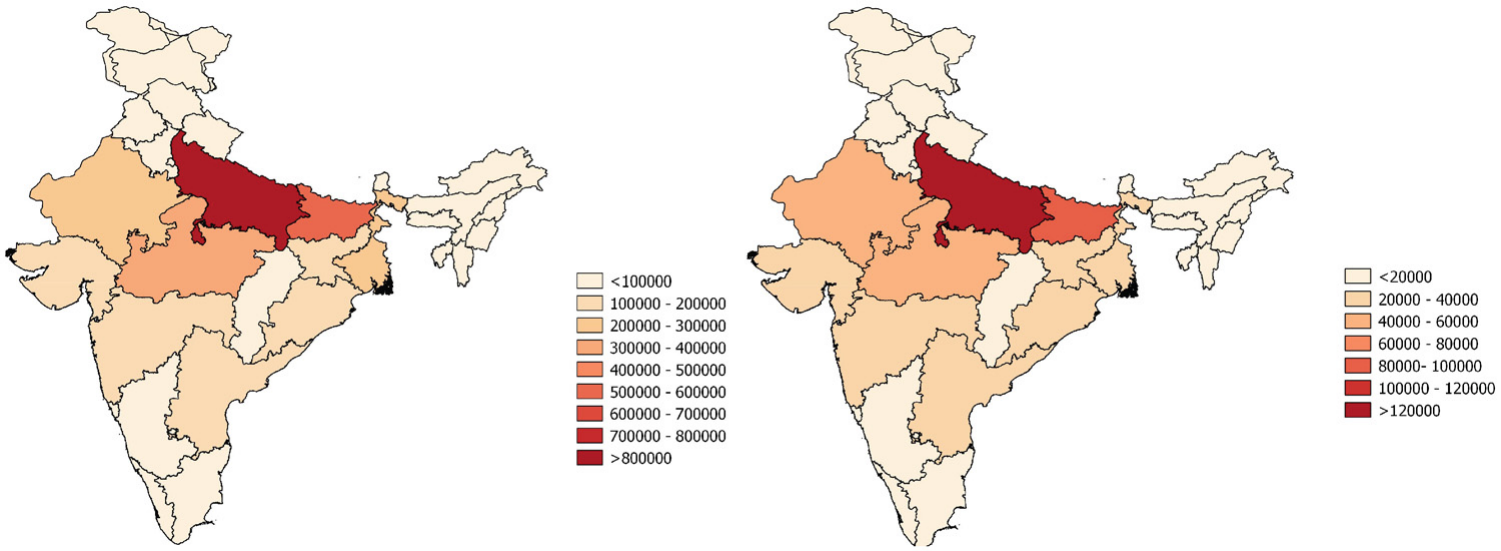
Distribution of severe pneumonia episodes and pneumonia deaths in children younger than 5 years in India. (A) Number of severe pneumonia episodes in children aged 0–59 months (B) Number of pneumonia deaths in children aged 0–59 months.

We estimated that Jharkhand had the highest incidence of severe pneumonia but Uttar Pradesh (UP) had the highest number of cases because of a large population of children younger than 5 years. The states that merit special mention include Uttar Pradesh (UP) from central India where 18.1% children reside but contribute 24% of pneumonia cases, Bihar (eastern India, 11.3% children, 16% of cases), Madhya Pradesh (MP) (central India, 6.6% children, 9% of cases), and Rajasthan (northern India, 6.6% children, 8% of cases). We observed that the distribution of pneumonia mortality among across Indian states followed a trend similar to that of severe pneumonia (Fig 1A). Of 3.6 million episodes of severe pneumonia and 0.35 million deaths that occurred in children aged less than 5 years in India, the top five contributors were UP (24% of cases and 26% of deaths), followed by Bihar (16% of cases, 22% of deaths), MP (9% of cases, 12% of deaths), Rajasthan (8% of cases, 11% of deaths) and Jharkhand (5% of cases, 6% of deaths) as top five contributors (Fig 1B).

**Fig 2:**
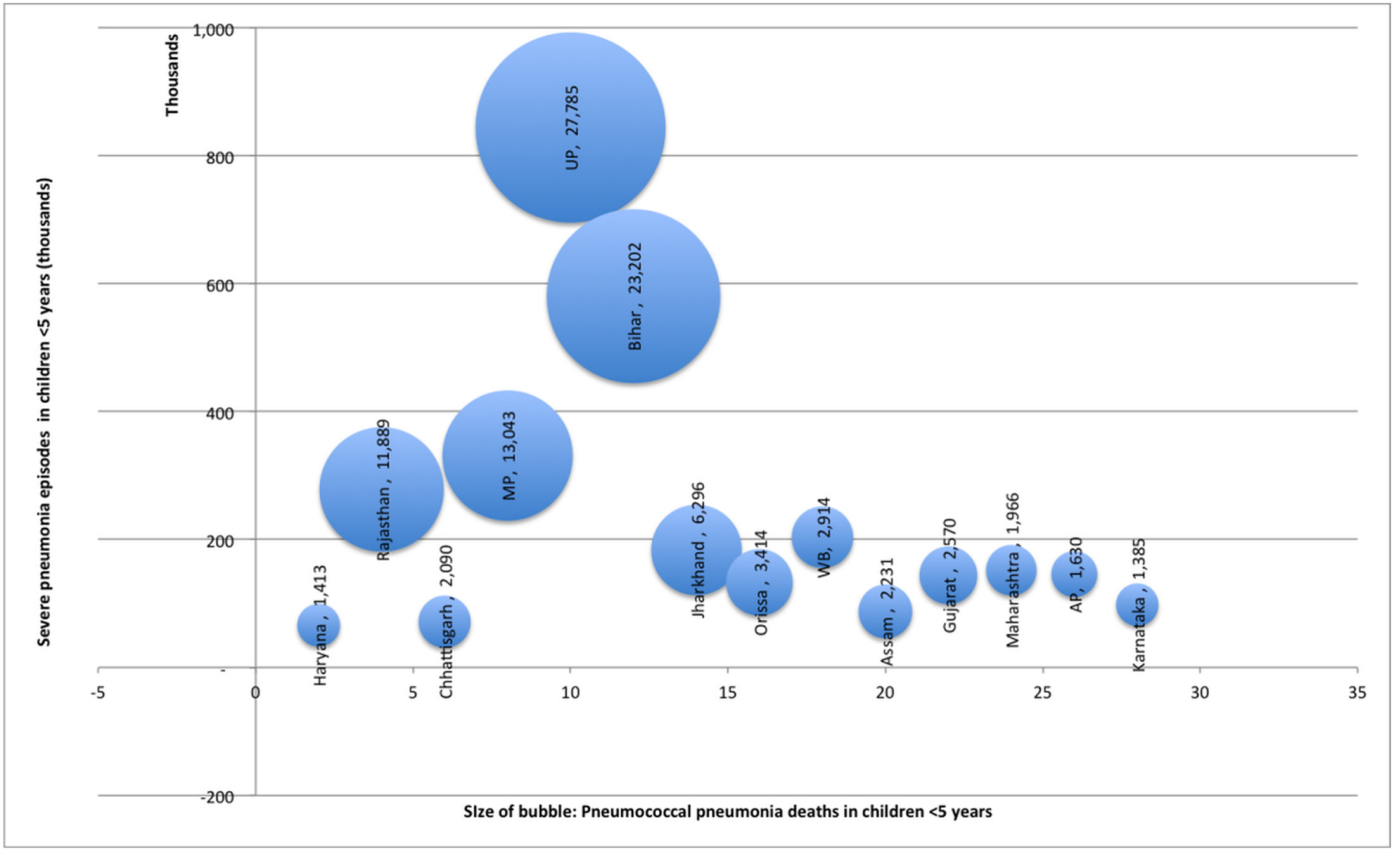
Selected Indian states with the highest number of pneumococcal pneumonia deaths in children younger than 5 years in 2010. Bubble size indicates the number of pneumococcal pneumonia deaths.

Further, we estimated that in year 2010, 0.56 million (0.49–0.64 million) severe pneumococcal pneumonia episodes and 105 thousand (92–119 thousand) pneumococcal pneumonia deaths had occurred in children younger than 5 years of age in India. The annual incidence of severe pneumococcal pneumonia in India was estimated to be 4.8 episodes (95% CI, 4.2–5.5) per 1000 children younger than 5 years. The top five contributors to India’s pneumococcal pneumonia burden in terms of number of cases and deaths were again Uttar Pradesh (1,33,167 cases 27, 785 deaths), Bihar (91,578 cases, 23,202 deaths) Madhya Pradesh (52,250 cases, 13,043 deaths), Rajasthan (43,911 cases, 11,889 thousand death) and Jharkhand (28,969 cases, 6296 deaths) (Fig 2). The incidence estimate for pneumococcal pneumonia episodes was highest in Jharkhand (7.9 episodes per 1000 child) and lowest in Manipur (1.1 episodes per 1000 child)

It is recognized that severe pneumonia has a higher proportion of cases attributable to *S*. *pneumonia*, and leads to more severe outcomes. It is thus preferred for use in estimating the burden of pneumonia. We also observed that the burden of clinical pneumonia was significantly higher than severe pneumonia: of all clinical pneumonia cases, 21.2% (95% CI, 18.0–24.9) were classified as severe, and we estimated that the proportion of pneumococcal pneumonia among all severe pneumonia was 15.8% whereas pneumococcal pneumonia deaths among all cause pneumonia deaths were 29.5%. Finally, we estimated a case fatality rate in severe pneumonia of 9.96%, comparable to case fatality rates of 10.8% reported in earlier estimates[8].

## Discussion

It is recognized that interpretation of country level pneumonia estimates is challenging, especially for large countries like India and China because clinical pneumonia incidence, access to care and childhood mortality vary substantially within the country[5]. We have attempted to address this limitation by generating state specific estimates on burden of severe pneumonia, pneumococcal pneumonia and pneumonia related mortality. Our pneumonia estimates are based on the epidemiological concept of potential fraction of key risk factors for pneumonia. Hence, our estimates capture the impact of varying levels of prevalence of key risk factors across different states in India. Also, varying degree of access to care across states has been captured in the model (S1 File). Further, the pneumococcal pneumonia estimates are based on applying a vaccine probe approach to an existing trial (see methods). The primary reason for adopting this approach is recognition of the fact that until new diagnostic methods become available, models using the vaccine probe approach are reliable in estimating cause-specific pneumonia disease burden[5].

To check the consistency and plausibility of severe pneumonia estimates generated by our model we compared them against other published estimates for India. We estimated 3.6 million episodes of severe pneumonia in children younger than 5 years for the year 2010, which was similar to the estimate of 4.0 million episodes reported by CHERG for India [8]. Further, our model predicted severe pneumonia incidence of 30.7 episodes per 1000 child per-year, which was similar to incidence reported by the CHERG (32.0 episodes per 1000 child per-year) [8]. The methodological and modelling approach adopted by CHERG group and our group were essentially the same. They key difference was in the parameter values as explained in methodology section. We also checked the plausibility of state-specific estimates for pneumococcal pneumonia. Our model predicted a pneumococcal disease incidence of 4.4 (95% CI, 3.8–5.0) cases per 1000 in children younger than 5 years of age. This was similar to an invasive pneumococcal disease rate of 4.5 cases per 1000 children reported from neighboring geographical area of Bangladesh by a population-based, active-surveillance, and active-case detection study[25].

In the state-specific analysis we observed that in general, the states having a high prevalence of pneumonia risk factors and poor access to health services had a higher burden of pneumonia cases and deaths[17, 18]. This is consistent with reports of successive national health surveys highlighting that poor health infrastructure and health services, poor percentage of institutional deliveries, low immunization coverage rates and other health system weaknesses have led to high pneumonia burden in these states[17, 18, 20, 26] especially UP, Bihar, MP and Rajasthan.

With reference to the contribution of pneumococcal pneumonia to severe pneumonia episodes, we estimated a pneumococcal pneumonia contribution of 15.8% to all-cause severe pneumonia, as compared to 18.4% reported by Rudan et al. [7, 8]. This difference possibly occurred because of differing methodological approaches for the estimation of attributable proportion. Within the framework of the vaccine probe approach, other researchers (Rudan et al, O’ Brien et al and Walker et al.) have used the conjugate vaccine efficacy against WHO-defined clinical pneumonia as a measure of the proportion of pneumonia cases attributable to *S*. *pneumoniae*[5]. Our group has used the conjugate vaccine efficacy against WHO-defined chest-radiography-positive pneumonia as a measure of the proportion of pneumonia cases attributable to *S*. *pneumonia*, with adjustment for vaccine serotype distribution across the India. Finally, our estimate of contribution of *S*. *pneumoniae* mortality (29.5%) to all cause pneumonia mortality is similar to that estimated by other researchers (32.9%)[7, 8].

Our national and state-specific estimates were generated using the same statistical model based on the epidemiological concept of potential impact fraction. Hence, the comparability of our national level estimates with other studies provides reasonable confidence that state level estimates for severe pneumonia, pneumococcal pneumonia and pneumonia deaths are also robust. Furthermore, we are reasonably confident of our age-specific estimates of pneumonia morbidity and mortality. We observed that the distribution of pneumonia cases and deaths followed a right skewed distribution with the highest burden in the youngest age group, and it then decreases in older groups[15]. Our findings are consistent with community based longitudinal cohorts and multi-centric surveillance studies from India reporting skewed distribution of pneumonia burden [12, 13, 22, 24, 27, 28].

One of methodological limitations of our model is that it does not necessarily assume that the risk factors are independent, because we applied a meta-estimate of relative risks derived primarily from the studies of multivariate design[29]. Also, our child population estimates were for the year 2010, the prevalence of exposures to risk factors was for the years 2006–07, and the childhood pneumonia estimates were based on studies conducted between 2007–2010. Hence, the estimates for pneumonia may be an over-estimate because the prevalence of risk factors has decreased, and access to care has increased over the last decade[7, 8]. Another potential factor that could contribute to an overestimate is availability of pneumococcal conjugate vaccines (PCV) in India. Although the PCV is available in the private sector since 2008, the coverage is negligible (<1% in major states) and the vaccine has not yet been introduced into the universal immunization programme.[30]

Another potential limitation of our study is the fact that other researchers assume that individual estimates of cause-specific etiologies for pneumonia add up to a total estimated pneumonia envelope because we have not attempted to do the same[7, 8]. The focus of our study was to offer insights into the actual distribution of severe pneumonia, pneumococcal pneumonia and pneumonia deaths to inform policy on pneumonia prevention and control. It is recognized that pneumonia related morbidity and mortality are good indicators of the economic burden of pneumonia, since households incur significant healthcare cost and productivity losses as a result of hospitalization due to pneumonia[31]. Information on the burden of severe pneumonia in different age groups and across all states in India would be helpful in designing and delivering targeted interventions to specific populations.

Finally, the use of conjugate vaccines against pneumonia, especially *Streptococcus pneumonia and Hemophilius influenza*, appears to be warranted for the prevention pneumonia morbidity and mortality in children younger than 5 years. In India, state specific estimates of the pneumonia burden and mortality could be used to identify states where vaccination and other interventions are required; and age specific estimates could help identify at what age vaccines should be used to achieve highest impact [15]. Adopting this practice would accelerate progress towards MDG4, and would also contribute to a reduction in regional disparities of child health indicators.

## Supporting Information

S1 File Parameterized Microsoft Excel based model. (XLS)
